# Theme recognition and evolutionary analysis of older adults cognitive disorder prevention and control policies based on the BERTopic model

**DOI:** 10.3389/fpubh.2026.1864997

**Published:** 2026-08-13

**Authors:** Wang Zili, Ji Huimin, Song Yulei, Luo Dan, Yin Tingting, Xu Guihua, Bai Yamei

**Affiliations:** School of Nursing, Nanjing University of Chinese Medicine, Nanjing, Jiangsu, China

**Keywords:** BERTopic model, cognitive impairment, older adults, policy text analysis, prevention and control

## Abstract

**Introduction:**

The early diagnosis and treatment of mild cognitive impairment (MCI) can help decelerate or even reverse the progression of dementia. Therefore, China attaches great importance to the prevention and control of MCI and has introduced a series of policies.

**Methods:**

Policy themes were identified by pre-processing, vectorizing, and clustering relevant policy texts by using the BERTopic model, and their temporal evolution patterns were revealed through dynamic topic modeling.

**Results:**

Central-level policies on cognitive impairment prevention and control encompass 12 themes, which can be classified into three major categories: community prevention and early screening, multistakeholder participation in rehabilitation work, and collaborative high-risk chronic disease management. Different work priorities were observed during the initial budding (2005–2018), progressive consolidation (2019–2023), and rapid development (2024–present) stages. Policies consider comorbidity management and demonstrate simultaneous systematization and refinement, with the focus of prevention and control gradually moving forward and responsibilities becoming increasingly diversified. However, issues, such as an underdeveloped comorbidity management system, insufficient coordination between central and local authorities, poor linkage between stages, and inadequate multistakeholder collaboration, persist.

**Discussion and implications:**

In the future, constructing cognitive impairment prevention and control implementation pathways tailored to local resources; establishing policy transition mechanisms; improving multidepartment and multistakeholder collaboration networks; and enhancing the role of personnel in early screening, rehabilitation interventions, and comorbidity management are necessary to promote effective policy translation and implementation.

## 1 Introduction

Dementia is a neurodegenerative condition characterized by acquired cognitive impairment, leading to a marked decline in individuals' daily living abilities and social interaction skills (1). China has approximately 17 million dementia cases, ranking first worldwide (2), with its number of cases continuing to rise. Currently, no effective cure for dementia exists. However, clinical studies indicate that early screening, diagnosis, and treatment during the early stage of dementia (3), specifically mild cognitive impairment (MCI), can reverse disease progression in 26% of individuals (4). Therefore, prevention and control measures during the MCI stage are crucial for dementia management. Given dementia's characteristics, such as public awareness gaps (5) and high social stigma (6), current prevention efforts face numerous challenges, necessitating enhanced government leadership and demonstration roles. Consequently, the Chinese central government has implemented multiple policies since 2006 to promote the prevention and control of cognitive impairment. Policies serve as the foundation for practical work, with policy documents constituting core elements (7). The focus of cognitive impairment prevention policies evolves dynamically over time; failure to identify current priorities promptly may lead to the misallocation and waste of human and material resources. Existing research reveals that strained community resource allocation and inefficiencies result in the underidentification of potential cases, thereby exacerbating the burden on healthcare systems (8). Most studies have examined the implementation status and effectiveness of cognitive impairment prevention policies from a real-world perspective, whereas only a few have explored policy-driven approaches to prevention efforts. This situation creates challenges in evaluating the alignment between practical implementation and policy frameworks through the dual lenses of real-world practice and theoretical guidance. Textual analysis of policy content is rarely performed in research, with existing studies largely focusing on overall national strategy (9). However, this analysis failed to assess the overall development trajectory of initiatives for cognitive impairment prevention. Current research remains limited in understanding the current focus and evolutionary stages of prevention policies. Technical topic identification aims to extract representative and forward-looking research themes from massive textual datasets with mainstream models, including word bag and neural topic models (10). This study employed the BERTopic model to conduct topic mining and temporal evolution analysis on central-level policies for cognitive impairment prevention, revealing underlying policy logic and developmental patterns to inform the establishment of nursing-oriented prevention systems.

## 2 Materials and methods

### 2.1 Data sources

This study focused on centrally issued policy documents regarding cognitive impairment prevention and control. Keywords, such as “cognitive disorder,” “dementia,” “cognitive impairment,” “mild cognitive impairment,” “senile dementia,” “prevention and control,” “intervention,” “screening,” were used for title matching searches. The search period covered January 1, 2005, to September 28, 2025. Search channels included the National Health Commission, the website of the Central People's Government, China's CNKI government documents, and the Peking University Fabao Database. After duplicates were removed, 41 policies were extracted, with some policies listed in Table 1.

**Table 1:** Summary of national-level policies on the prevention and treatment of cognitive disorders in China (excerpt).

Order number	Policy name	Release time	Publisher
1	Notice on issuing the development outline of the national key basic research program (973 program) for the 11th 5-year plan	2006-10-25	Ministry of Science and Technology of the People's Republic of China
2	Notice on issuing the development outline of the national science and technology support program for the 11th 5-year plan	2007-02-13	Ministry of Science and Technology of the People's Republic of China
3	Notice on issuing the national mental health work system development guidelines (2008–2015)	2008-01-15	National Health Commission of the People's Republic of China
...	...	...	...
39	Notice on the implementation of the year of action for pediatric and mental health services (2025–2027)	2025-04-28	National Health Commission of the People's Republic of China
40	Notice of the general office of the national health commission on issuing the guidelines for the construction and management of geriatrics departments (2025 edition)	2025-05-08	National Health Commission of the People's Republic of China
41	Notice of the general office of the national health commission on prohibiting the application of deep cervical lymphatic node–vein anastomosis in the treatment of Alzheimer's disease	2025-07-08	National Health Commission of the People's Republic of China

### 2.2 Research methods

**Figure 1:**
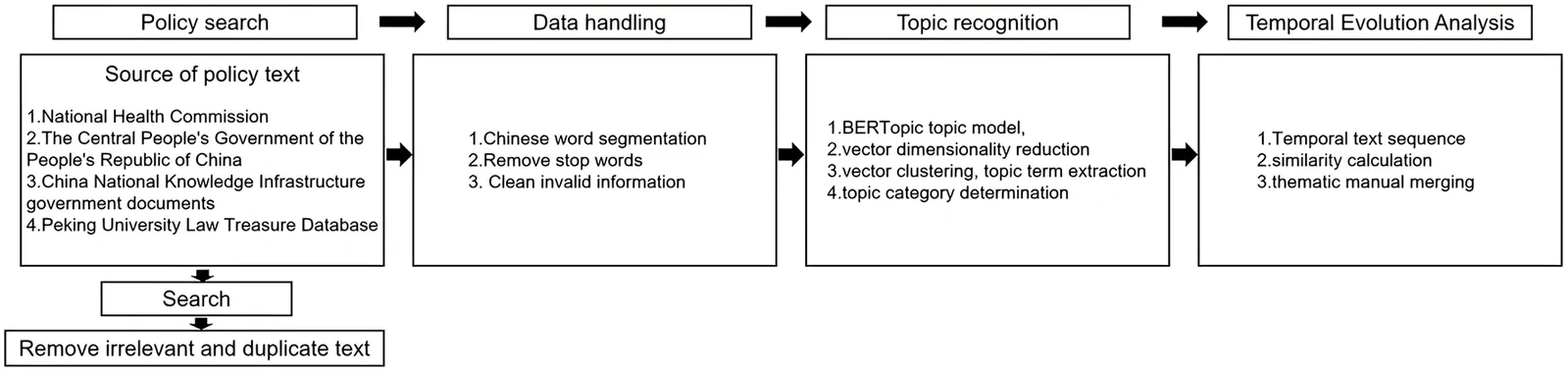
Technical roadmap for policy theme analysis related to cognitive impairment.

This study, which used Python 3.11 software for data analysis and constructed a BERTopic model for data processing, comprised the following steps: first, standardization processes, including word segmentation, keyword filtering, dictionary loading, and stop word removal were applied to transform raw policy texts into short-text structured data suitable for model processing. Second, the pre-trained BERT model performed vectorization on processed texts to extract deep semantic features. Third, semantic representation–based clustering analysis was conducted to identify topic categories preliminarily. hierarchical merging was implemented through hierarchical clustering methods to consolidate initial topics progressively until a final set of significantly differentiated themes was obtained. The technical roadmap of this study is illustrated in Figure 1. Finally, following the establishment of thematic clusters, the Dynamic Topic Modeling (DTM) procedure under the BERTopic architecture is deployed with annual time intervals. The refinement of topic characterizations, achieved via fine-tuning and global optimization, serves to facilitate a rigorous diachronic analysis of the developmental trajectories inherent in policy topics.

## 3 Results

### 3.1 Overall topic recognition

**Figure 2:**
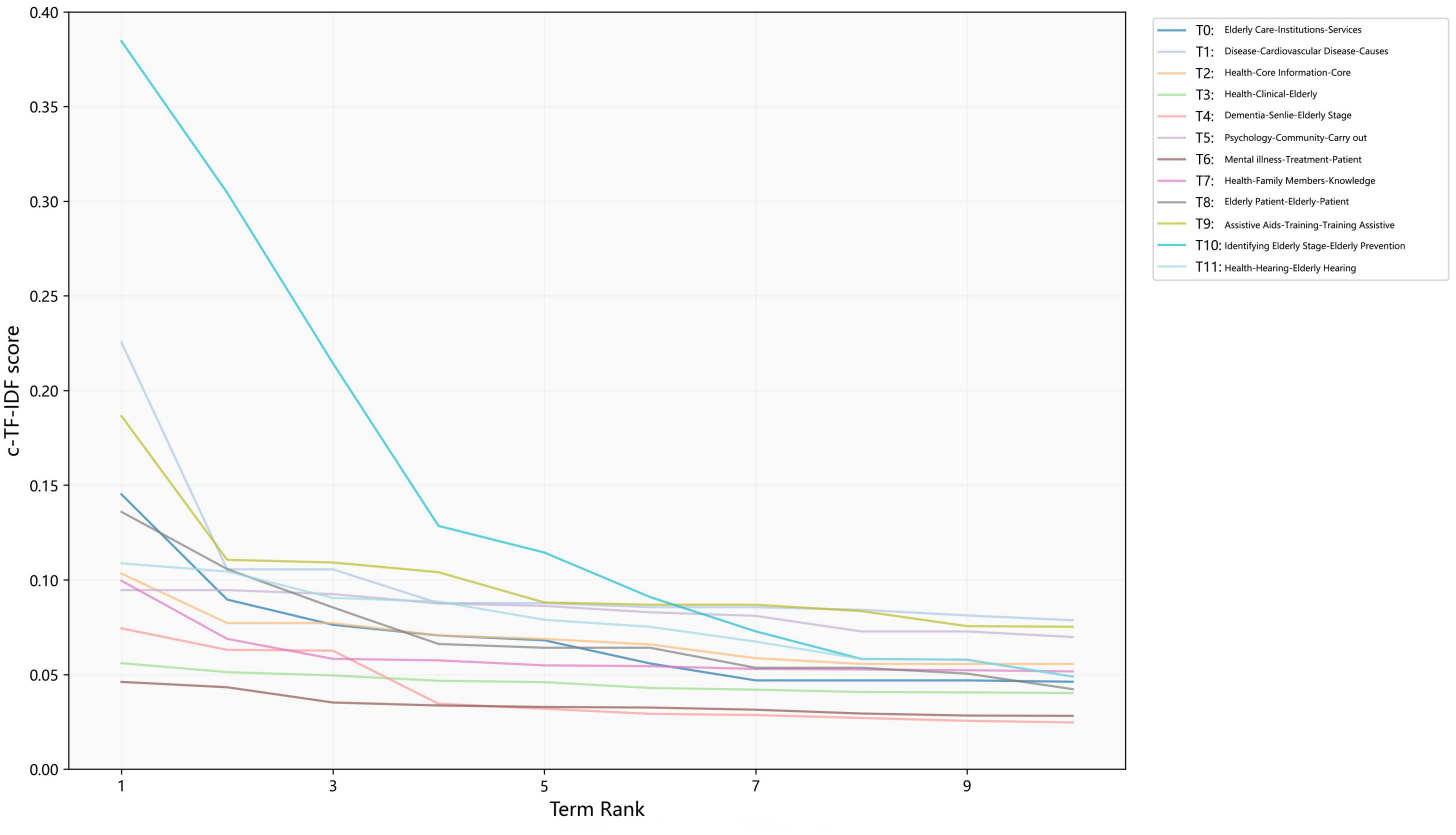
Trend of feature word weight decline.

**Figure 3:**
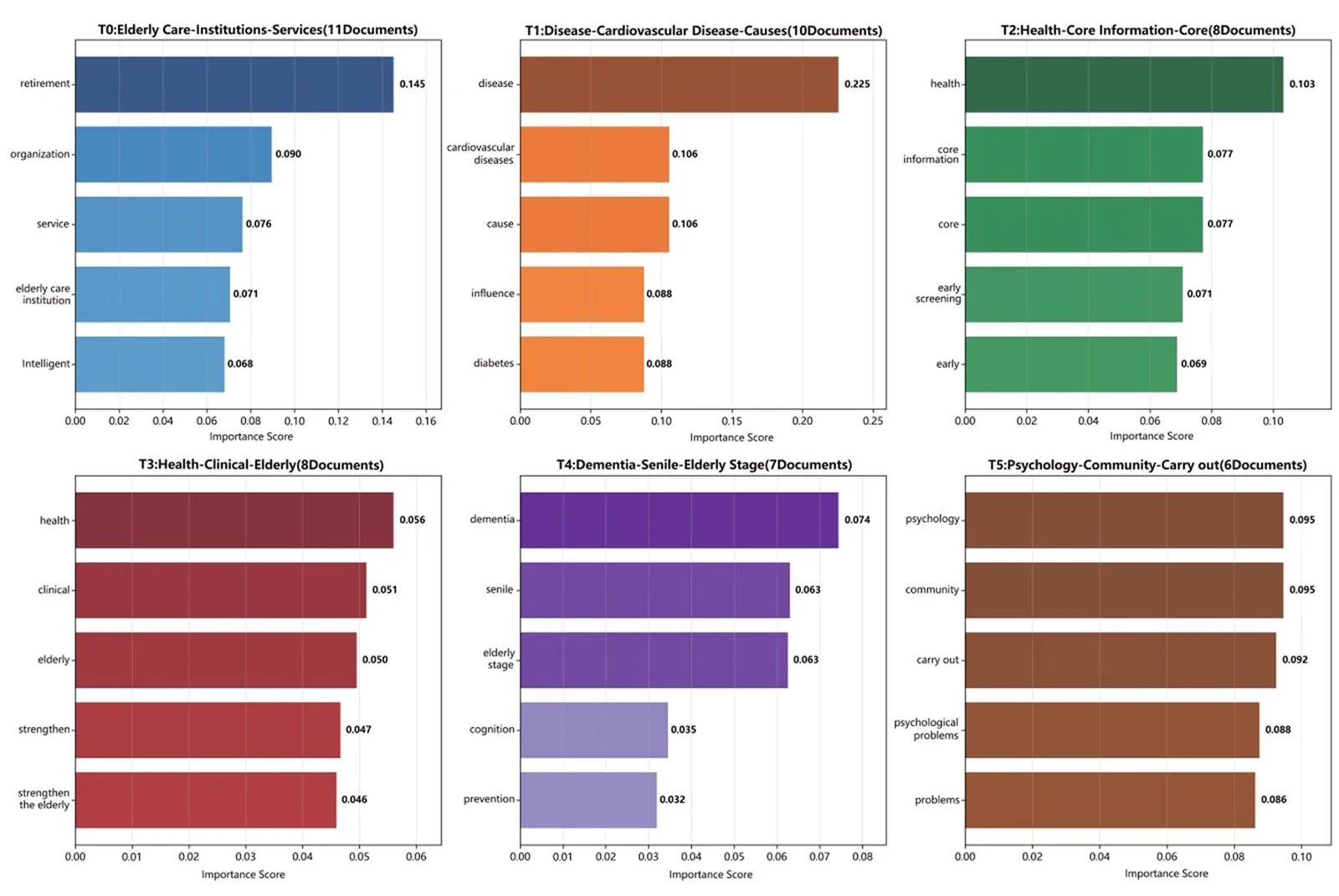
Key thematic terms and their weights in the relevant policy section.

**Figure 4:**
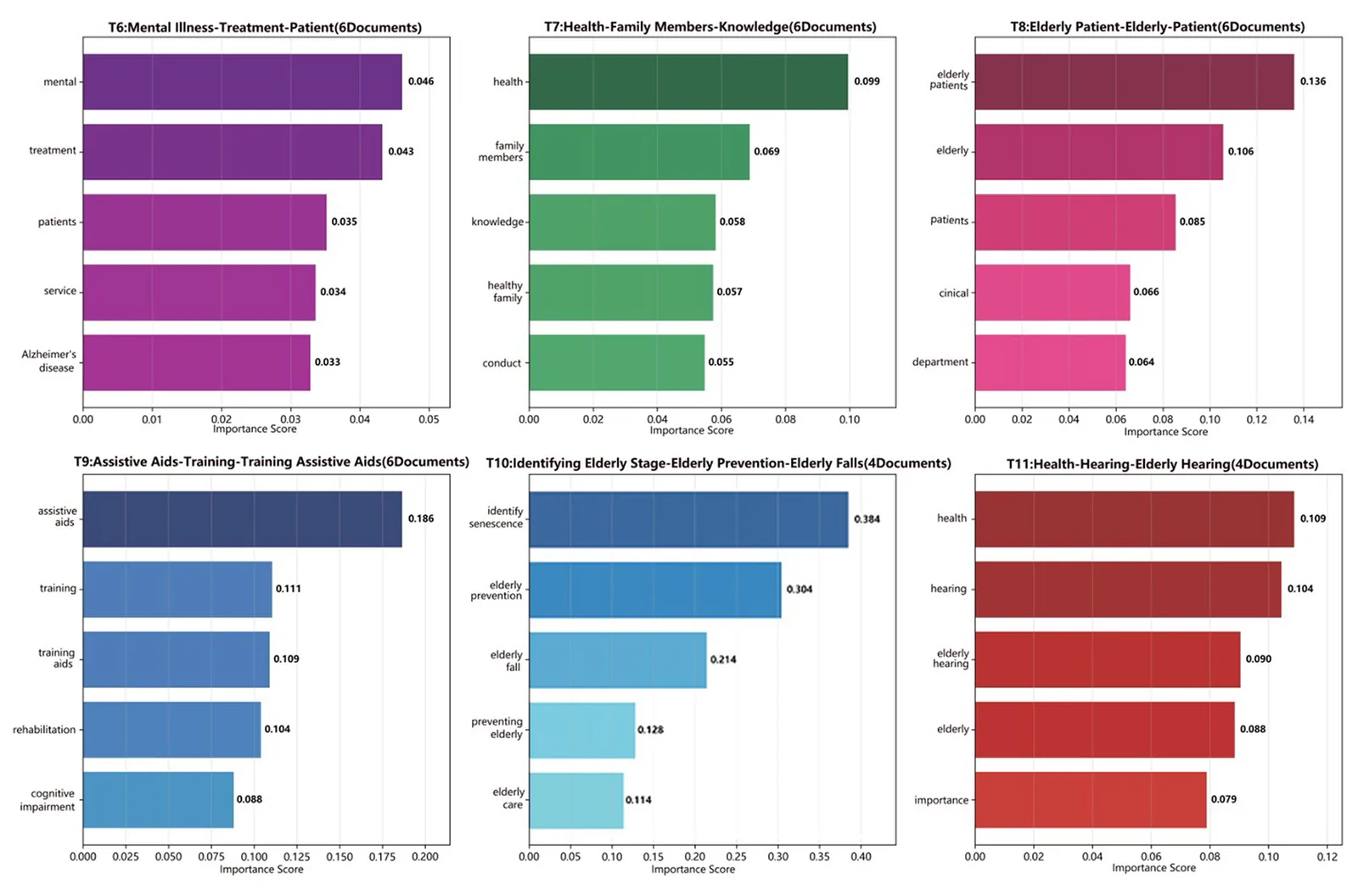
Key thematic terms and weights of relevant policies.

The BERTopic model was employed to conduct topic identification on policies related to cognitive impairment, automatically generating 12 thematic clusters. The analysis of feature word weight trends across these clusters (Figure 2) revealed that most topics required 3–5 characteristic terms for effective representation, without significant improvement observed beyond six terms. By building on this foundation, the model was iteratively refined (Figures 3, 4), with high-weight feature words utilized to describe topics, such as Topic 0 (older adults care institutions and services). The model uses UMAP with number of neighbors = 5, HDBSCAN with minimum cluster size = 2, and ngram_range = (1, 2). The model achieved a silhouette coefficient of 0.79, exceeding the generally accepted threshold of 0.5. In the robustness assessment, the Adjusted Rand Index (ARI) ranged from 0.76 to 0.85, and the Normalized Mutual Information (NMI) was consistently above 0.92, indicating a high level of cluster consistency. The Jaccard similarity coefficient for topic words ranged from 0.58 to 0.71, reflecting a moderately high degree of overlap. This suggests that the semantic cores of the topics are stable, with variations primarily confined to peripheral vocabulary. Sensitivity analysis of dimensionality reduction with respect to random seeds yielded an ARI above 0.90 and a Jaccard coefficient above 0.84. The topic coherence score (C_v) was 0.45.

**Figure 5:**
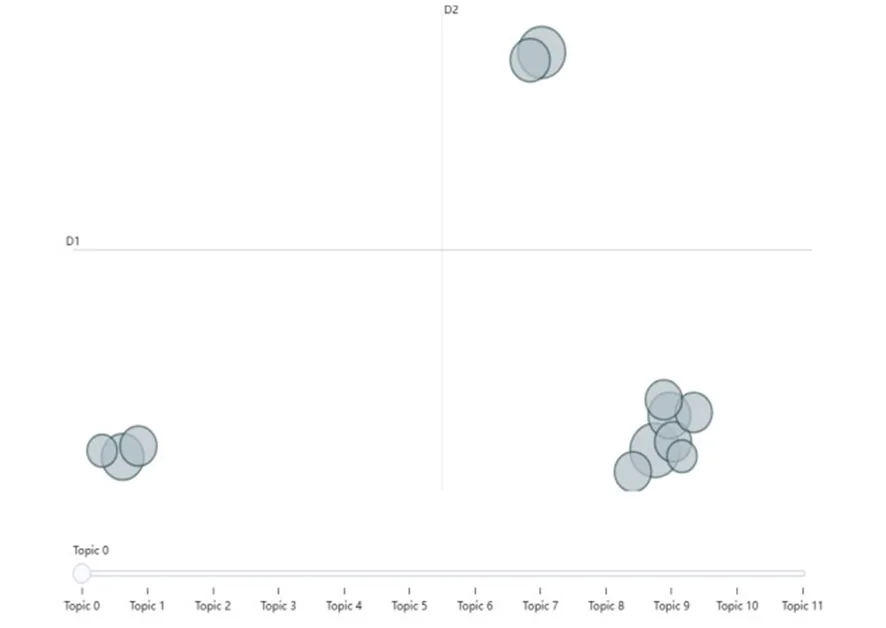
Distribution of thematic categories.

**Figure 6:**
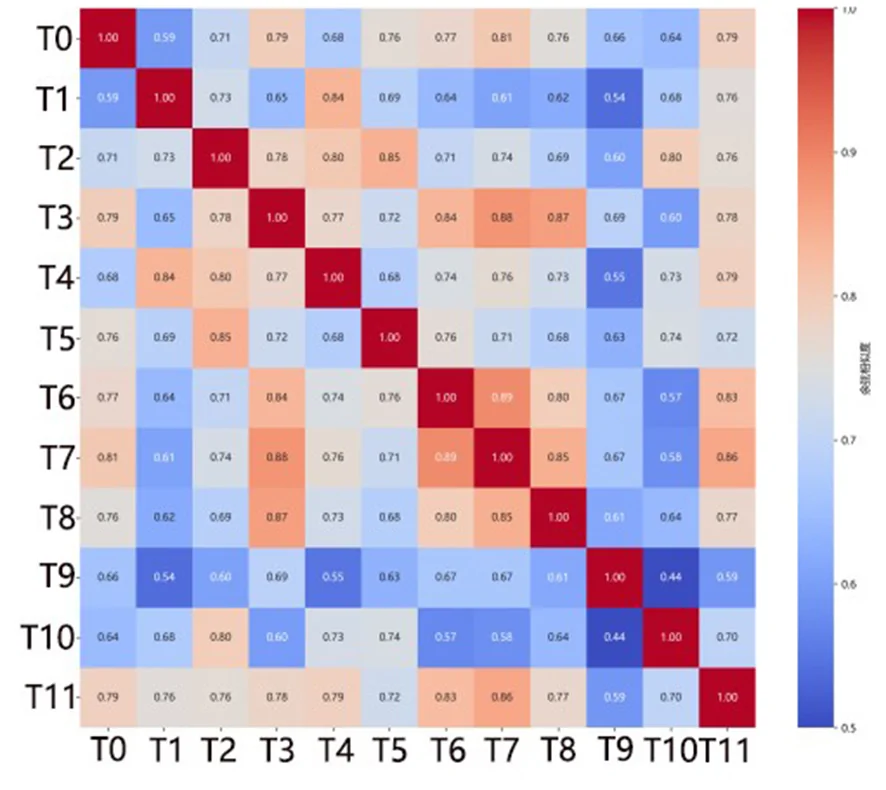
Cosine similarity heatmap.

Further hierarchical clustering was conducted by using topic category distribution and cosine similarity (Figures 5, 6), aggregating the 12 topics into three core categories with high internal consistency and interclass discriminability. Cluster analysis evaluation demonstrated a clear and rational topic classification structure. Through integrated clustering analysis and manual interpretation, three major thematic categories for cognitive impairment prevention and control policies were ultimately established: community-based prevention and early screening, multistakeholder participation in rehabilitation efforts, and Lack of Specialized Screening Services (Table 2).

**Table 2:** Theme cluster categories.

Order number	Topic category	Theme	Keywords (top 20)
1	Community prevention and early screening	Topic 2 (Health_Core Information_Core); Topic 5 (Psychology_Community_Implementing Older adults Care); Topic 10 (Identifying Aging_Preventing Falls in the Older adults)	Identification of aging; prevention in the older adults; fall prevention in the older adults; care for the older adults; falls; care; identification; aging; prevention; health; psychology; community; implementation of older adults care; psychological issues; problems; mental health; geriatric psychology; core information; core
2	Multistakeholder participation in rehabilitation efforts	Topic 0 (Older adults Care_Institutions_Services); Topic 3 (Health_Clinical_Older adults); Topic 6 (Mental Illness_Treatment_Patient); Topic 7 (Health_Family Members_Knowledge); Topic 8 (Older adults Patients_Older adults_Patients); Topic 9 (Assistive Aids_Training_Training Assistive); Topic 11 (Health_Hearing_Older adults Hearing)	Assistive aids; older adults care; geriatric patients; training; training assistive devices; health; aging; hearing; rehabilitation training •Health; older adults hearing; institutions; aging; cognitive impairment; promotion; assistive devices; robots; patients; rehabilitation; importance; services
3	Lack of Specialized Screening Services	Topic 1 (Disease_Cardiovascular Disease_Causes); Topic 4 (Dementia_Senile_Older adults Stage)	Disease; cardiovascular and cerebrovascular diseases; cause; impact; diabetes mellitus; society; Alzheimer's disease; older adults disability Hearing; decline; dementia; senile dementia; older adults; cognitive; prevention and control; dementia prevention and control; older adults individuals; diagnosis and treatment; function; training

#### 3.1.1 Community prevention and early screening

Communities serve as a critical frontline for identifying individuals with cognitive impairment. Given the current lack of an effective cure for Alzheimer's disease, early detection and intervention during the reversible stage of MCI are pivotal for slowing or even reversing disease progression. Cluster analysis revealed that current policies prioritize early prevention and screening efforts in communities, focusing on three key areas: early screening for older adults populations (Topic 2), psychological issue identification in older adults (Topic 5), and comprehensive early prevention measures (Topic 10). This finding indicates that policy design not only emphasizes MCI screening but also integrates mental health conditions prevalent among the older adults, such as depression, that are strongly associated with the risk of Alzheimer's disease, thereby establishing a holistic early defense system.

#### 3.1.2 Multistakeholder participation in rehabilitation services

The topic clustering results revealed that late-stage intervention and rehabilitation for cognitive impairment in China are primarily advanced through collaborative efforts by multiple stakeholders, with core participants including older adults care institutions (Topic 0), medical institutions (Topic 3), patient families (Topic 7), older adults individuals themselves (Topic 8), and assistive device development enterprises (Topic 9). Additionally, as key implementers, hospitals and older adults care institutions extend their responsibilities beyond cognitive impairment itself to encompass the diagnosis and treatment of related mental disorders (Topic 6) and associated fields, such as hearing interventions (Topic 10). This finding indicates that China's cognitive impairment prevention and control efforts, while clarifying the core responsibilities of each participant, further expand the scope of disease management to build an integrated and efficient prevention and control network.

#### 3.1.3 Lack of specialized screening services

Cluster analysis showed limited specialized cognitive function screening services, with most being managed in a mixed manner with chronic diseases, such as cardiovascular diseases and diabetes (Topic 1). This dependent service model has caused cognitive screening to lack independent positioning and dedicated resource support within primary public health service systems. Given its incomplete separation from chronic disease management, cognitive screening lacks specialized design in service content, implementation processes, and quality control, making achieving the early identification and precise management of high-risk populations difficult. Consequently, this situation hinders the overall advancement of dementia prevention and control efforts.

### 3.2 Temporal evolution analysis of policy themes

**Figure 7:**
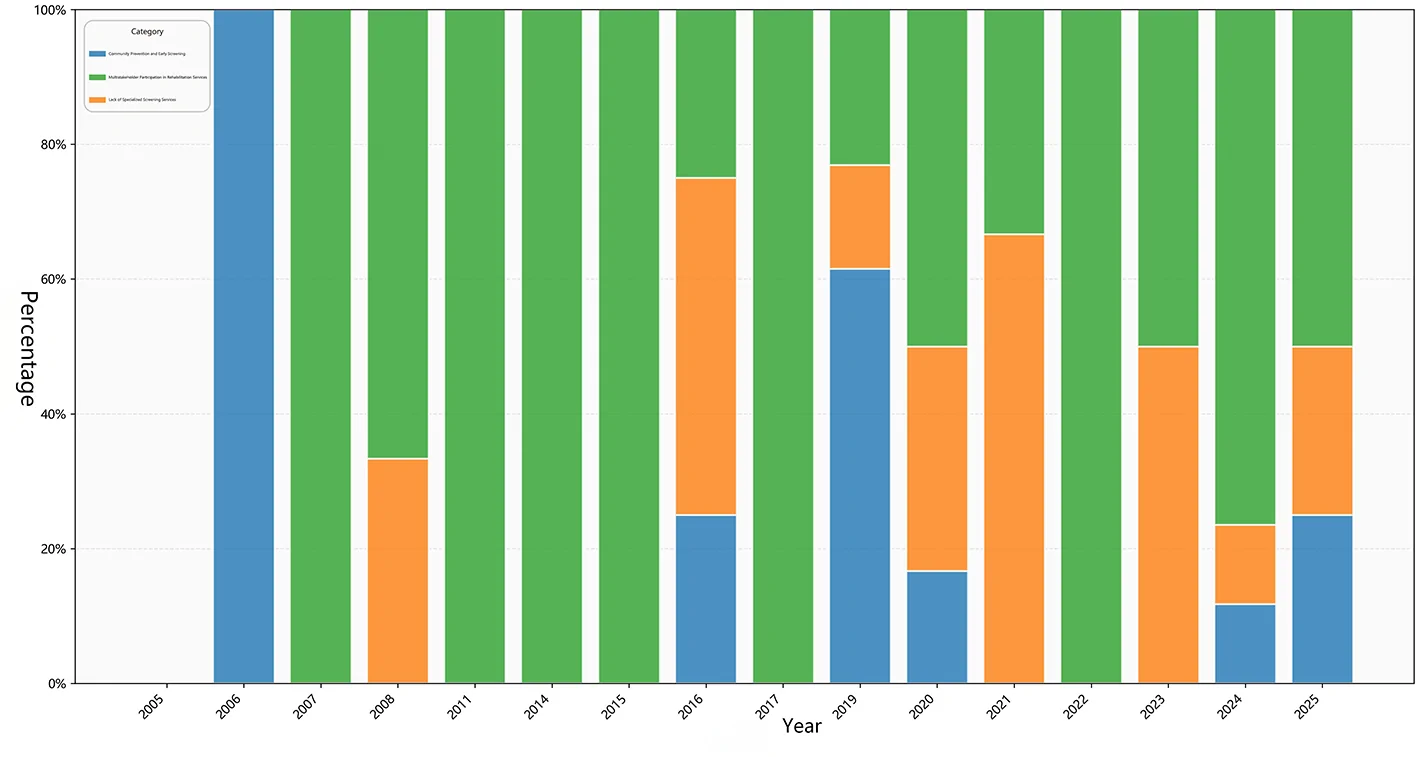
Temporal proportion trends of each thematic category.

**Figure 8:**
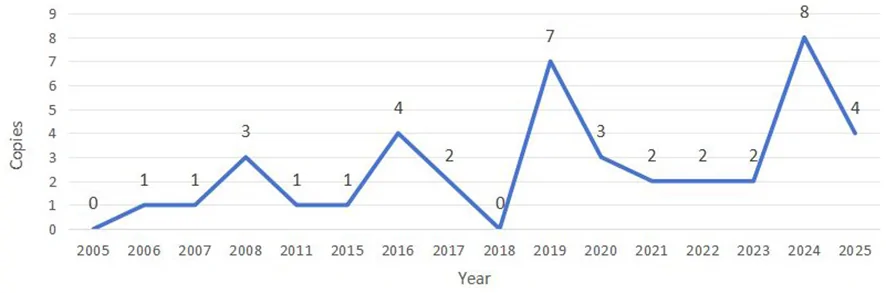
Historical distribution of policy quantity by year.

The temporal evolution of three topics intuitively reflects the developmental trends of core themes in China's cognitive impairment prevention and control policies (Figure 7). As shown in Figure 7, early-stage policies focused on community-based prevention and early screening, whereas the primary policy objectives initially centered on the collaborative management of high-risk chronic diseases. After 2016, policy priorities diversified, with multistakeholder participation in rehabilitation initiatives receiving increasing attention. Notably, community prevention and early screening categories peaked in 2019 and 2025. The annual policy quantity distribution is illustrated in Figure 8.

On the basis of theme identification, temporal evolution, and policy distribution results, the policies can ultimately be categorized into three phases: the initial budding (2005–2018), advanced consolidation (2019–2023), and rapid development (2024–present) stages.

#### 3.2.1 Initial budding stage

During this phase, policies on cognitive impairment prevention and control were in their nascent stage. They pre-dominantly consisted of macrolevel guiding statements without systematic frameworks tailored to specific risk populations, scenarios, or implementation pathways. Their key characteristics included limited policy volume and absent standalone specialized policies, with therapeutic focus concentrated on advanced disease stages, such as Alzheimer's disease. Between 2005 and 2018 (the study period), only 13 related policies were issued. Among these, Lack of Specialized Screening Services accounted for the highest proportion. Most were incorporated into broad mental health or geriatric health policy frameworks, failing to recognize cognitive impairment as an independent and urgent public health issue requiring dedicated attention. The substantial disease burden imposed on older adults individuals, families, and society was also inadequately addressed in policy formulation. Furthermore, policies primarily focused on treating older adults individuals in the irreversible stage of Alzheimer's disease rather than establishing disease management systems for reversible cognitive impairment phases. This situation, to some extent, hindered the optimal allocation of public health resources throughout the disease lifecycle.

#### 3.2.2 Advanced consolidation phase

This stage witnessed a rising proportion of Multistakeholder Participation in Rehabilitation Services, implying that the top-level structure of the dementia prevention and control system was becoming more complete over time. Policies related to cognitive impairment have rapidly expanded in response to the goal proposed in the Healthy China Initiative (2019–2030) (11) of reducing the growth rate of dementia prevalence among individuals aged 65 years and above by 2030. A total of 16 policies were introduced within 5 years, accounting for 39% of the overall policy framework. During this phase, policy development became increasingly systematic, establishing a preliminary three-tier prevention network encompassing etiological prevention, “three early” prevention (early detection, early diagnosis, and early treatment), and clinical prevention or disease management (12). The focus of prevention and control systematically shifted to the reversible MCI stage prior to dementia onset. Concurrently, collaborative prevention models integrating the government, professional institutions, communities, and families were explored. This series of top-level designs and systematic frameworks provided clear and actionable guidelines for local practices. Since 2019, cities, such as Beijing and Shanghai, have actively responded to central directives (13, 14) by initiating pilot programs for cognitive impairment prevention and control. These local efforts, through multistakeholder collaboration with tertiary hospitals, communities, and older adults care institutions, have led to the establishment of cognitive impairment–friendly communities to coordinate comprehensive prevention and control efforts.

#### 3.2.3 High-speed development stage

At present, policy efforts are still largely dominated by “Lack of Specialized Screening Services,” yet for two consecutive years, they have focused on community-based prevention and early screening. Moreover, since 2024 the frequency of policy releases in the field of cognitive impairment prevention and control reached a new high, with policy focus further shifting toward community-level interventions and increasingly detailed policy content. By building upon the established three-tier prevention system, this phase saw the systematic reinforcement of top-level design for community-based prevention and early screening through the intensive issuance of specialized documents. Enhanced disease awareness campaigns markedly increased the strategic weight of public health education, early prevention in high-risk populations, and early cognitive function screening in the older adults within overall prevention strategies. Moreover, the 2025 National Action Plan for Addressing Age-Related Dementia (2024–2030) issued detailed implementation objectives (15), specifying targets for disease awareness, initial screening, and medical consultation rates. This plan provided local governments with a clear action blueprint, fundamentally driving the transition of grassroots prevention efforts from task execution to standardized, goal-oriented operational models.

## 4 Discussion

### 4.1 Cognitive impairment prevention and control policies trend toward systematization and refinement

Research findings indicate that China's cognitive impairment prevention and control policies have evolved through three stages from initial inception to rapid development, with their systematic and refined trends prominently reflected in two dimensions: system construction and target specification. In terms of system construction, policies have established a three-tier prevention network encompassing etiological prevention, “three early” interventions (early detection, early diagnosis, and early treatment), and clinical prevention or disease management (12). Implementation tools and protocols, such as educational videos, screening miniprograms, and non-pharmacological interventions, have been provided to primary care settings through measures like encouraging development, recommending usage, or direct allocation (16). Regarding target specification, policy objectives have progressed from vague early recommendations to clearly defined regulations with explicit expectations. Currently, no studies have focused on the overall developmental trends of cognitive impairment–related policies. This deficiency reflects the ongoing optimization of prevention and control strategies. With the continuous rise in the prevalence of MCI, cognitive impairment has increasingly become a major public health challenge for the nation, prompting responsive adjustments in relevant policies. However, despite the establishment of a comprehensive top-level framework, policy implementation still faces multiple constraints: First, at the execution level, practical barriers, such as insufficient disease knowledge dissemination, low healthcare-seeking willingness among older adults populations, and shortages in specialized facility allocation (8), exist. Second, at the structural level, China's medical resource distribution exhibits considerable regional imbalance and spatial concentration characterized by dense concentrations in eastern regions and sparse distribution in western areas (17). This resource disparity further leads to the prioritization of limited resources for urgent disease prevention and control objectives by public health decision-making in resource-scarce regions, thereby constricting the funding space for cognitive impairment prevention. This situation makes the single top-level prevention framework face promotion challenges because it struggles to adapt to regional resource allocation disparities and lacks practical feasibility for resource-deficient areas as a result of overly prescriptive targets. Therefore, subsequent optimization efforts should focus on establishing actionable and targeted implementation pathways to implement policies effectively. Refining prevention systems in accordance with local conditions while maintaining core principles; designing region-specific prevention strategies aligned with resource capabilities; and setting phased, geographically tailored, and achievable objectives are recommended to facilitate policy implementation effectively.

### 4.2 Gradual shift in policy focus for cognitive impairment prevention and control

On the basis of theme identification and temporal evolution analysis, this study reveals that the focus of cognitive impairment prevention policies has progressively shifted earlier in stages. During the initial budding phase, prevention efforts primarily concentrated on clinical treatment for individuals with advanced-stage diseases, such as Alzheimer's disease. In the consolidation phase, emphasis gradually shifted toward early intervention for MCI, with the preliminary establishment of a three-tier prevention system and attention to infrastructure development, including dementia-friendly communities and cognitive service centers (18, 19). During the rapid development phase, focus further advanced to community-based prevention and early screening, encompassing public education initiatives, high-risk population interventions, and cognitive function assessments of the older adults. Overall, policies demonstrate a clear trend of shifting focus from late-stage disease treatment to early-stage prevention. This evolution aligns with the epidemiological characteristics of cognitive disorders, enhancing resource allocation efficiency, and strengthening the scientific rigor of prevention strategies. However, phased policy shifts may also lead to implementation challenges and short-term operational disruptions. Local governments are advised to establish transitional coordination mechanisms during policy implementation to address the above issue, clarifying responsibilities and workflows during transitional periods to reduce execution uncertainties caused by policy shifts. This approach ensures the smooth transition of practical operations and effectively alleviates staffing burdens at grassroots levels.

### 4.3 Increasing the diversification of implementing entities for cognitive impairment prevention and control policies

With the gradual improvement in the cognitive impairment prevention and control system, its project operation model has evolved from a single government-led approach to a collaborative governance framework involving multiple stakeholders, including governments, tertiary hospitals, community health service centers, subdistrict offices, older adults care institutions, and social organizations. Since the issuance of the Notice on Exploring Specialized Services for Depression and Alzheimer's Disease Prevention and Treatment in 2020 (20), medical institutions at all levels, older adults care facilities, and qualified social organizations have progressively participated in cognitive impairment prevention and control efforts, leveraging their respective professional strengths to form synergistic collaboration. Specifically, tertiary hospitals primarily undertake standardized diagnosis and treatment tasks for geriatric dementia, establishing referral and consultation linkage mechanisms with primary healthcare institutions to guide follow-up management (21). Older adults care institutions focus on providing day care services, short-term residential care, cognitive training, and emergency rescue services for individuals with dementia while accelerating the development of dedicated dementia care zones within these facilities (12). Social welfare organizations are committed to disease awareness campaigns and organizing antiwandering initiatives to foster a social environment that respects, understands, and cares for individuals with dementia. The multistakeholder collaborative mechanism has achieved systematic progress in cognitive impairment prevention and control across diagnostic services, community management, and social support systems, considerably enhancing overall prevention and control efficacy. However, despite effective operations within their respective professional domains, challenges, such as the unclear delineation of responsibilities and inefficient mechanisms of coordination, persist in overlapping areas with ambiguous jurisdictional boundaries. Further improving multiparty collaboration models in the field of cognitive impairment prevention and control, with integrated medical and older adults care services and physician–community coordination being examples of such models, is recommended. Promoting close collaboration among multiple stakeholders by clarifying the division of responsibilities, establishing coordination mechanisms, and enhancing information sharing is essential. This approach will facilitate the systematic integration of resources and maximize the synergistic efficacy of all participating parties in cognitive impairment prevention and control.

## 5 Summary

This study employed the BERTopic model to conduct topic identification and evolutionary analysis on cognitive impairment prevention and control policies issued at the central level from 2005 to 2025. It systematically mapped the policy evolution trajectory and development trends in this field in China. It identified three distinct phases in the evolution of central-level cognitive impairment prevention policies: the initial budding (2005–2018), progressive consolidation (2019–2023), and rapid development (2024–present) stages. Overall, the policies demonstrated a trend defined by the integration of comorbidity management with systematic and refined approaches, with prevention priorities gradually shifting forward and responsibility becoming increasingly diversified. Future efforts should focus on establishing localized cognitive impairment prevention models to enhance the effectiveness of policy implementation. By building upon top-level policy design, tailored implementation pathways should be developed on the basis of regional resource conditions and practical needs, ensuring stratified, categorized, and actionable strategies to achieve tangible outcomes in cognitive impairment prevention and control initiatives.

The study has the following limitations. First, dementia prevention and control efforts in China are still in their early stages, and the number of policy documents available for analysis is relatively limited, which may introduce some bias into the results of the thematic modeling. Second, this study focuses primarily on national-level policies and does not include text analysis of specific implementation policies at the provincial and municipal levels; therefore, it is difficult to conduct an in-depth comparison of the differences in policy development and actual implementation across different regions. Future research could further expand the sample of policy documents by incorporating policies for the prevention and control of cognitive impairment at different levels and across different regions into the analytical framework. This would allow for an exploration of the top-down transmission and implementation effectiveness of policies, as well as a comparison of regional differences in implementation, thereby providing more robust empirical support for optimizing the tiered and categorized implementation of policies.

## References

[1] Tang L. A study of Screening Intentions and Screening Behaviors of People at High Risk for dementia Based on the Theory of Planned Behavior. Guangzhou: Jinan University (2023).

[2] Wang G Qi J Liu X Ren R Lin S Hu Y . China Alzheimer report 2024. Diag Theory Pract. (2024) 23:219–56. doi: 10.16150/j.1671-2870.2024.03.001

[3] Jia L Quan M Fu Y Zhao T Li Y Wei C . Dementia in China: epidemiology, clinical management, and research advances. Lancet Neurol. (2020) 19:81–92. doi: 10.1016/S1474-4422(19)30290-X 31494009

[4] Qin Y Han H Li Y Cui J Jia H Ge X . Estimating Bidirectional transitions and identifying predictors of mild cognitive impairment. Neurology. (2023) 100:e297–307. doi: 10.1212/WNL.0000000000201386 36220593 PMC9869761

[5] Alzheimer's Society. World's largest dementia study reveals two thirds of people still incorrectly think dementia is a normal part of ageing, rather than a medical condition[EB/OL]. London: Alzheimer's Society (2019). Available online at: https://www.alzint.org/news-events/news/worlds-largest-dementia-study-reveals-two-thirds-of-people-still-incorrectly-think-dementia-is-a-normal-part-of-ageing-rather-than-a-medical-condition (Accessed September 8, 2025).

[6] Nimmons D Manthorpe J West E Rait G Sampson EL Iliffe S . Views of people living with dementia and their carers on their present and future: a qualitative study. BMC Palliat Care. (2023) 22:38. doi: 10.1186/s12904-023-01165-w 37032342 PMC10084652

[7] Zhang B Li P Chen J Guo Q Wu YR. Thematic analysis and evolutionary process of national science and technology innovation policies: a perspective based on text mining. Sci Stud Sci Technol Manag. (2019) 40:15–31. doi: 10.20201/j.cnki.ssstm.2019.11.002

[8] Wang C Wang X Zhang L Wang L. Advances in community screening and diagnostic management strategies for individuals with mild cognitive impairment. China Gen Pract. 1–8. Available online at: https://link.cnki.netUrlid/13.1222.R.20230810.1051.006 (Accessed December 22, 2025).

[9] Tan Z Jing Q Duan M. Policy text analysis of cognitive impairment in the elderly in China under the policy tool theory. China Health Manag. (2023) 40:557–60.

[10] Huang H Wang CY. Analysis of technological evolution, development trends and innovation ecosystem of generative AI in China based on BERTopicand long short-term memory networks. Sci Technol Manag Res. (2025) 45:178–90.

[11] National Health Commission of the People's Republic of China. Healthy China Initiative (2019–2030). Beijing: National Health Commission (2019). Available online at: https://www.nhc.gov.cn/guihuaxxs/c100133/201907/2a6ed52f1c264203b5351bdbbadd2da8.shtml (Accessed December 15, 2025).

[12] Wang Y Sun L Wang G Li C Zhou H Du Y . Community-based three-level comprehensive prevention and treatment of Alzheimer's disease: China expert consensus (2025 Edition). Chinese J Geriatr. (2025) 44:227–37. doi: 10.3760/cma.j.issn.0254-9026.2025.03.001

[13] Beijing Municipal Health Commission. Notice on issuing the implementation plan for the Phase I project of elderly brain health examination (dementia risk screening) in Beijing. (2019) Available online at: https://wjw.beijing.gov.cn/zwgk_20040/ghjh1/201912/t20191216_1242718.html (Accessed 15 December, 2025).

[14] Shanghai Municipal People's Government. Notice on Issuing the Implementation Plan for Deepening Elderly Care Services in Shanghai (2019–2022). Huangpu: Shanghai Municipal People's Government (2019). Available online at: https://www.shanghai.gov.cn/nw44392/20200824/0001-44392_59184.html (Accessed 15, December, 2025).

[15] The The Central People's Government of the People's Republic of China National Health Commission. Notice on Joint Issuance of the National Action Plan for Addressing Senile Dementia (2024–2030) by the National Health Commission and 15 Other Departments[EB/OL]. Beijing: Central People's Government (2025). Available online at: https://www.nhc.gov.cn/lljks/c100158/202501/4b4476877f21467fbfcea71bc90d8313.shtml (Accessed December 15, 2025)

[16] Department Department of Aging Health National Health Commission. The Department of Aging Health, National Health Commission released the 2025 World Alzheimer's Month Thematic Publicity Campaign Toolkit [EB/OL]. (2025). Available online at: https://www.nhc.gov.cn/lljks/c100157/202509/c7f7bc64b2eacb50220147ae441.shtml (Accessed December 15, 2025).

[17] Yin S Liu Z Yu S Li Y An J Wang D . Geographic variations, temporal trends, and equity in healthcare resource allocation in China, 2010–21. J Glob Health. (2025) 15:04008. doi: 10.7189/jogh.15.04008 39819771 PMC11737812

[18] Lu Y Liu C Wells Y Yu D. Challenges in detecting and managing mild cognitive impairment in primary care: a focus group study in Shanghai, China. BMJ Open. (2022) 12:e062240. doi: 10.1136/bmjopen-2022-062240 36127116 PMC9490618

[19] Shi J Wang Z Zhang X Song Y Xu G Bai Y. The current status of initial cognitive screening services in community-based cognitive services centers in Nanjing. China Gen Prac. (2025) 28:2784–90.

[20] The Central People's Government of the People's Republic of China. Notice of the General Office of the National Health Commission on Exploring Specialized Services for Depression and Alzheimer's Disease Prevention and Treatment[EB/OL] (2020). Available online at: https://www.gov.cn/zhengce/zhengceku/2020-09/11/content_5542555.html (Accessed December 15, 2025).

[21] Feng C Chen Y Wang W Chen S. Can medical consortiums bridge the gap in health inequity in China? A propensity score matching analysis. Health Policy Plan. (2025) 40:727–36. doi: 10.1093/heapol/czaf031 40434033 PMC12360172

