# Supplementary material for: Theme recognition and evolutionary analysis of older adults cognitive disorder prevention and control policies based on the BERTopic model
Source: Front Public Health. 2026 Aug 13;14:1864997. doi: 10.3389/fpubh.2026.1864997 (PMC13518341; doi:10.3389/fpubh.2026.1864997)
Supplement: Supplementary file 1 [file Table_1.DOCX]

**1.Definition of the BERTopic model:** It is a dynamic modeling framework that integrates modules such as pre-trained models, word vector dimensionality reduction, and clustering. The model combines the powerful word embedding techniques of bidirectional encoder representations from transformers (BERT) with clustering algorithms, capturing contextual semantic information of text through high-dimensional embeddings. Compared with traditional methods (e.g., LDA), BERTopic can capture fine-grained contextual relationships and generate more accurate and interpretable topic representations.

**2.The BERTopic model typically performs topic mining through three steps:** text embedding, topic dimensionality reduction and clustering, and topic representation.

Text embedding: After data preprocessing, a pre-trained language model is used to convert each policy document into a high-dimensional semantic vector, capturing the deep meaning of the document.

Topic dimensionality reduction and clustering: UMAP is applied for dimensionality reduction, and then the HDBSCAN algorithm groups semantically similar documents into the same topic cluster.

Topic representation: c-TF-IDF is used to extract the most representative keywords/phrases for each topic cluster.

**3.Reasons for selecting the BERTopic model in this study:**

① BERTopic has become a well‑established analytical tool in policy research. A substantial body of literature has demonstrated its effectiveness in policy text mining, with applications spanning multiple policy domains such as artificial intelligence, information security, energy, and public welfare.

② The most fundamental breakthrough of BERTopic lies in its shift from "statistical co‑occurrence" to "semantic understanding." Traditional LDA models are based on the bag‑of‑words assumption, treating documents as collections of individual words and inferring topics solely from word co‑occurrence frequencies, without capturing contextual meaning. For example, although "AD" and "Alzheimer’s disease" refer to the same concept, LDA may assign them to different topics unless manual mapping or substitution is performed during preprocessing.

③ This study focuses on policies related to cognitive dysfunction at the central level of China. These policies simultaneously involve multiple stages—disease prevention, clinical intervention, rehabilitation care, and social security—and are jointly formulated or separately issued by various authorities, including health, industry and information technology, and civil affairs. Due to overlapping stages and intersecting responsibilities among the issuing bodies, the governance themes implicitly embedded in these policy texts are interwoven and blurred. The BERTopic model can automatically identify these core governance themes, quantify the weight of each theme within the overall policy system, and support temporal analysis of thematic evolution, thereby systematizing and normalizing what were originally mixed and ambiguous topics.

**4.Detailed Explanation of the Preprocessing Section**

①Text Source

Using Python's urllib and selenium libraries, policy data were scraped from the official websites of national, provincial, and prefectural governments and their departments. A total of 1,016 policy documents were retrieved. After removing 529 duplicates, 398 documents were excluded by reading the titles, and another 48 were excluded after full‑text review, resulting in 41 currently effective policies. As shown in Figure 1 .

Figure 1 Policy Screening Flowchart

②Key text selection

Read the 41 relevant texts, select key sentences and paragraphs, remove irrelevant content such as data sources, page numbers, and special characters, while ensuring that the input text length is below 512 tokens. The tools used for Chinese word segmentation and stopword filtering are listed in Table 1.

Table 1 Word Segmentation Tool Selection

| Function | Library Name |
| --- | --- |
| Chinese word segmentation | jieba word segmentation library |
| Stopword filtering | Harbin Institute of Technology stopword list + custom policy stopwords |

③Bertopic model dependency toolkit

Table 2 Bertopic model dependency toolkit

| Function | Library Name |
| --- | --- |
| Core Tool | bertopic>=0.15.0  sentence-transformers>=2.2.0  umap-learn>=0.5.3  hdbscan>=0.8.29 |
| NLP related (text processing) | jieba>=0.42.1  scikit-learn>=1.0.0 |
| Data Processing | pandas>=1.3.0  numpy>=1.21.0  openpyxl>=3.0.0 |
| Visualization | matplotlib>=3.5.0  seaborn>=0.11.0  plotly>=5.10.0 |
| Other | tqdm>=4.62.0 |
